# Male mating biology

**DOI:** 10.1186/1475-2875-8-S2-S8

**Published:** 2009-11-16

**Authors:** Paul I Howell, Bart GJ Knols

**Affiliations:** 1 Centers for Disease Control and Prevention (CDC), 4770 Buford Hwy, Atlanta GA USA 30341, Malaria Research and Reference Reagent Resource Center (MR4) and Atlanta Research & Education Foundation (AREF), 1670 Clairmont Road (151F), Decatur, GA 30033, USA; 2 Div. Infectious Diseases, Tropical Medicine & AIDS, Academic Medical Center, F4-217, Meibergdreef 9, 1105 AZ Amsterdam, The Netherlands and K&S Consulting, Kalkestraat 20, 6669 CP Dodewaard, The Netherlands

## Abstract

Before sterile mass-reared mosquitoes are released in an attempt to control local populations, many facets of male mating biology need to be elucidated. Large knowledge gaps exist in how both sexes meet in space and time, the correlation of male size and mating success and in which arenas matings are successful. Previous failures in mosquito sterile insect technique (SIT) projects have been linked to poor knowledge of local mating behaviours or the selection of deleterious phenotypes during colonisation and long-term mass rearing. Careful selection of mating characteristics must be combined with intensive field trials to ensure phenotypic characters are not antagonistic to longevity, dispersal, or mating behaviours in released males. Success has been achieved, even when colonised vectors were less competitive, due in part to extensive field trials to ensure mating compatibility and effective dispersal. The study of male mating biology in other dipterans has improved the success of operational SIT programmes. Contributing factors include inter-sexual selection, pheromone based attraction, the ability to detect alterations in local mating behaviours, and the effects of long-term colonisation on mating competitiveness. Although great strides have been made in other SIT programmes, this knowledge may not be germane to anophelines, and this has led to a recent increase in research in this area.

## Background

Currently, control of malaria vectors focuses on the use of insecticide-treated bed nets and indoor residual spraying. Due to the rapid emergence and spread of insecticide resistance, alternative methods are needed to control vector populations. Many of these technologies, such as entomopathogenic fungi and viral paratransgenesis, have already shown great promise in laboratory settings but still have to be evaluated under field conditions [1-3].

An additional vector control method currently being reconsidered is the sterile insect technique (SIT). SIT is based on the concept of releasing sterilised, but sexually competitive males who will mate with wild females who thus lay sterile eggs which reduces vector populations and hence reduces or halts disease transmission [4]. Since the overall competitiveness of released vectors is expected to be below that of the wild insects and to accomplish an effect rapidly, an excess of sterile males relative to the number in the field have to be released. Although there have been many successes with other dipterans, earlier mosquito SIT programmes often produced conflicting results ([5] and [6]). Many failures have been linked to a lack of understanding of male mating biology, especially in regard to the ability of the mass-reared males to seek and mate with wild females [7]. Failures to control *Culex tarsalis*, *Culex tritaeniorhynchus *and *Anopheles culicifacies *were all correlated with inadequate knowledge of male mating behaviour [8].

Understanding mating in the context of the local vector population is likely to be paramount for success. Inferences about mating behaviours are limited as most of the information known is derived from studies using a limited number of tropical and temperate species. Very little is known about how and where males and females meet and what effect colonisation has on mating behaviours. The fundamental lack of knowledge in mating biology highlights the need to conduct research into how anophelines locate, identify, compete for and secure matings with conspecifics.

## Male sexual maturation

There is a paucity of information available on the sexual behaviour of male anopheline mosquitoes. Most information is 30 years old and derived from research done mainly with colonised *An. culicifacies, Anopheles gambiae*, or *Anopheles stephensi*. From these early studies an incomplete picture of male sexual development and mating biology can be pieced together. Males are not competent to mate at emergence since terminalia, sexual organs and antennal fibrillae must first mature. During the first 12-24 h after emergence the male's terminalia undergo an inversion of 180° in order to be properly oriented for mating [9]. Even though terminalia inversion is typically completed in 24 h, males are still not sexually mature as reflected by a delay in sexual activity. Spermatogenesis is reported to begin in the late pupal stage [10,11], however mating is not dependent on the presence of mature spermatozoa alone. Peak spermatogenesis in *An. stephensi *occurred between 0-5 days post emergence [12], however males became sexually active 48 h post emergence with peak mating activity 3-7 days post emergence [13]. Although *An. gambiae *and *Anopheles arabiensis *males were sexually active 48 h post-emergence, males younger than three days old had low rates of insemination [14] peaking seven days post emergence [15,16]. Mating in laboratory-reared *An. gambiae *was associated with the accumulation of male accessory gland (MAG) proteins even though sperm were present in the vesicles at 28 h post emergence [15]. Accumulation of MAG proteins requires 72-100 h [15,17] and was noted to be coincident with increased mating activity in male anophelines [12,13,15].

Additionally, full function of the male antennal fibrillae which is essential for mating does not occur until 12 h post emergence. Erect fibrillae respond to female flight tones and are required for location of females [9,18]. Three to four day old male *An. stephensi *were most responsive to female wing beat sounds [19]. Sexually mature male *An. gambiae *mosquitoes that had one or both their antennae removed were unable to locate females to mate [14,19]. Receptivity to wing beat tones, therefore, appears to be concordant with sexual maturation in males.

## Mate location

Mating in most anopheline mosquitoes is often believed to take place only in swarms [20,21] although a recent report suggests indoor mating in African anophelines [22]. Marchand [23] hypothesized that positive phototaxis in *An. gambiae *males occurs typically between 300-500 lux and primarily at dusk. Similar ranges have been observed in other anophelines. Light changes from 592 lux to 18.3 lux stimulated *An. culicifacies *males to mate in the laboratory [24] while in nature it was reported to be between 467.2 to 26.9 lux [25]. *Anopheles freeborni *males commenced swarming around 350 lux and dispersed around 0.5 lux [26]. The onset of mating is not related simply to the decrease in light intensity but is also tied to the inherent circadian rhythm in anophelines. Although lux values associated with mating occurred during the day, *Anopheles franciscanus *did not swarm until sunset [27]. Charlwood [14] showed a reduction in inseminations by males that had their circadian rhythm altered.

The interplay of circadian rhythm, swarming stimulation, and light level is reflected by the disparate lux values associated with the onset of mating. These interspecific differences may serve as mating barriers. In Pakistan, wild anophelines were found to commence swarming over the same markers but at different times [28]. However, in the laboratory, four members of the *An. gambiae *complex were found to have differing active periods with some overlap between sibling species [29]. Therefore, although swarming may be initiated at different times in some species, other mechanisms, such as spatial factors, must act to isolate species with overlapping swarming periods.

Swarms have been reported to form at varying heights as well, possibly determined by species-appropriate swarm markers. Markers are visually evident objects or contrasting areas either on the ground or horizon used for orientation during swarming [20]. There is some difficulty in associating specific markers with swarming males, or even being certain that they exist, as many natural ones are not evident to the observer. *An. gambiae *has been observed swarming between 2-3 m over no discernable marker [30] and 1-4 m [31] above ground markers. *An. gambiae s.l. *swarms were observed between 0.5-2 m with no discernable markers [23]. *Anopheles funestus *males formed swarms at heights of 2-4 m employing horizon markers [32]. Sympatric populations of *Anopheles hyrcanus *and *Anopheles philippenensis *swarmed at a height of 0.3-0.6 m above small shrubs and flowers (ground markers) and 1.5-2.5 m in large open spaces (horizon markers) [33].

Swarming height may also be related to orientation with swarm markers. Male S-form *An. gambiae *swarm near female feeding sites with an unobstructed view of the sky [23]. *An. funestus *swarms appeared to rely more on horizon markers [32] whereas *An. gambiae *and *Anopheles merus *relied more on ground and horizon markers [22,31,34]. Similarly, *An. freeborni *utilized both horizon and ground markers [26], and *An. culicifacies *males preferred to swarm over low mounds. Indoor-mating M-form *An. gambiae *oriented themselves in doorways and near eaves irrespective of any ground markings [22]. Swarming at different heights, therefore, may act as another barrier to prevent inter-species mating. Several genera of insects have been reported to swarm during the same period over the same marker [31], even then therefore, aggregations formed at varying heights could result in inter-species segregation.

Although much work has been done to elucidate what constitutes a swarm site, it has yet to be determined why males of different species choose certain areas. The need for a marker has been shown in several studies though none is evident in others. When a potential horizon marker was obscured, swarms of *An. funestus *were seen to gain altitude in order to re-orient themselves in the same place [32]. Similarly, swarms followed large cloth ground markers that were moved for short distances [34]. Potential swarm markers that were removed, resulted in the abandonment of that site by males [31]. In contrast, placing visual markers under swarms of *An. funestus *apparently led to displacement of the swarm (J.D. Charlwood, *pers. comm. *[32]).

Therefore, mosquitoes locate themselves in space and time to ensure they are available to mate. The interplay of time of initiation, marker type, and height in swarm formation reduce the probability of intraspecific mating [20]. Mixed species swarms are rare even in areas where sympatric species occur. In the field a limited number of heterospecific individuals have been collected during swarm sampling. The M- and S-forms of *An. gambiae *swarm at similar times, however they are rarely ever encountered in the same area [30,35]. Similarly, of 31 *An. gambiae *swarms sampled in Tanzania only five contained other anophelines [23]. Cunningham-van Someren routinely collected large swarms of *Anopheles squamosus *in Kenya that contained no more than one or two individuals of other anophelines [36]. The difference in marker recognition, resulting in intra-specific swarming, was shown between *An. gambiae *and *An. funestus *in Mozambique [32]. However, what constitutes an appropriate mating arena or what features are used for orientation for many species remains elusive.

## Swarming and mating behaviour of anophelines

**Figure 1:**
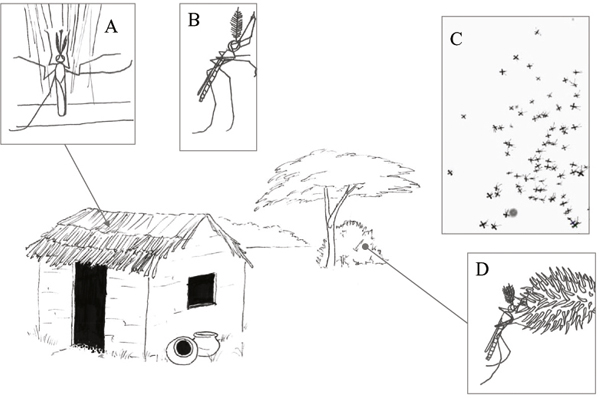
**The sexually mature male**: A. diurnal indoor resting, B. 1 hour prior to swarming the antennal fibrillae become erect, C. males depart to commence swarming, D. After swarming ceases, male ingest a sugar meal prior to resuming indoor resting.

Sexually mature male mosquitoes leave their diurnal resting sites at species specific times to commence swarming (Figure 1). The antennal fibrillae become erect and typically remain so for 1-2 h [14], however in laboratory-reared *An. stephensi *this period lasted up to six hours [37]. Swarming commenced at various times for different species [25,26,30,34]. As dusk nears, males begin to fly over the swarming arena [25,34]. Gradually more males join forming large, loose aggregations. Eventually the males move from a distended, non-specific circling motion into a more rigid, condensed group [25,34]. Swarms are comprised mainly of males (Table 1) with numbers varying from approximately 5-5000 individuals.

**Table 1:** Captured anopheline males and females from various wild swarms

**Species**	**males**	**females**	**max. % of females**	**Avg. number swarming**	**Reference**
*An. culicifacies*	715	5	7.0	nd	[24]
*An. culicifacies*	544	14	2.4	15-3000	[25]
*An. franciscanus*	2341	3	0.1	1000-5000	[27]
*An. freeborni*	6028	175	8.0	300-1500	[26]
*An. gambiae *(M)	2823	6	0.1	nd	[30]
*An. gambiae *(S)	511	6	4.0	nd	[35]
*An. squamosus*	611	0	0.0	40-300	[36]
*An. stephensi*	1061	18	2.8	nd	[59]
*An. subpictus*	2242	157	20.0	75-500	[98]

nd: No data given by author

Both in the laboratory and in the field, females become active after the males. Hence copulations are not usually observed until 5-20 min after swarm initiation. In Malaysia, *An. philippinensis *started mating 5 min after swarming commenced while *An. hyrcanus *began mating 10 min later [33]. *An. gambiae *and *An. funestus *males started mating approximately 10 min after swarming began [26] and *An. freeborni *commenced mating 5-10 min after swarming began. It is unknown why males appear at swarming arenas before females. Females may arrive later than males due to different phototactic stimuli. This may also be a behavioural response by males to ensure that they arrive at mating arenas before females to optimize their chances for mating.

**Figure 2:**
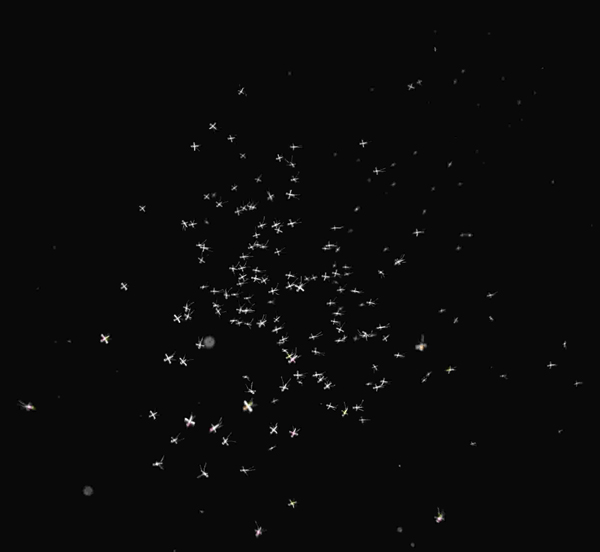
***Anopheles pharoensis *Theobald swarming (Photo courtesy of J.D. Charlwood)**.

Once formed, the swarm begins to move as a single unit (Figure 2). Male *An. gambiae *were observed to be unevenly distributed within the swarm with more males occupying the centre versus the periphery [38]. This centralized positioning may increase a males' chance to acquire a mate by either occupying the space most frequented by females or being more likely to better detect approaching females. It may also be a function of males aligning themselves with a potential swarm marker.

Swarming males have been reported to either mate with females as they penetrate the group [31,32,34] or as they temporarily departed to secure a female flying nearby [23,25,39]. Approaching females may be detected by their wing beat frequencies, which is typically lower than that of males. The range for detection is unknown, but in the laboratory *An. gambiae *males could detect a female at distances of 50-75 mm [14]. The ability of the male to detect conspecific female wing beats and reject heterospecific females has been proposed as another barrier to interspecific mating in *An. gambiae s.l. *[40]. Males of some mosquito species attempt to harmonize their wing beats with nearby insects (sexual tuning) to determine if they are conspecific and of the opposite sex [41] although this remains unknown for anophelines. Wild populations, however, display a greater variance in their tones resulting in widespread overlap and reduced discrimination of wing beat sound as a single way for males to identify conspecific females at a distance [42,43].

The actual period during which mating occurs in the swarm is brief. Once mating commences, copulating pairs are seen usually for 10-30 min as darkness approaches [30,32,35]. This shortened mating period may be related to the length of time spent *in copula*. Copulation in *An. gambiae *lasts 15-20 s [14] and approximately 27 s in *An. culicifacies *[25].

Detailed studies on copulation in *Anopheles *have shown that as a male approaches the female he grasps her with his tarsal claw on his first pair of legs and then swings his abdomen up to clasp her genitalia. Once interlocked, the male releases his tarsal grasp of the female and they assume the venter-to-venter position and resume flying [14,20,44]. The copulating pair then departs the swarm and flies off to complete the mating after which the male is assumed to rejoin the swarm. It is unknown if males will mate again on the same night, however observations exist of anopheline males returning to a swarm after mating (J.D. Charlwood, *pers. comm. *[26]).

Male courtship is considered to be absent in many swarming insects [45]. Instead, when females are a limiting factor and the costs of mate finding are high, males may forgo "choosy" behaviours so as to not waste potential mating opportunities [46]. This results in a "no-choice" scenario for both sexes as males cannot refuse a mate based on some phenotypic character and females will have limited choice, if any, in accepting a male's advances. There are no reported field observations of swarming males seeking out and rejecting a female. Possible indiscriminate mate choice by males was reported in wild *An. funestus*. Wing lengths of females caught in *copula *were not different from those of newly emerged females [32]. Therefore, males are likely to mate with females of unknown quality as their chances of meeting and copulating are low. In contrast, tethered virgin females have been reported to dislodge males attempting to copulate in the laboratory [14].

It remains unknown if males are capable of assessing female phenotypic quality and differentially allocate copulatory resources based on female quality during mating [46]. Due to low female availability, the reported high risk of predation during swarming, and the energy budget required for doing so, males will have limited opportunities to mate [21]. Therefore, it would benefit males to invest in quality inseminations thereby imposing monogamy on females via the insertion of a large volume of sperm and a mating plug. Natural behaviour corroborates this as polyandry is uncommon in wild *An. gambiae *(2.5%) (reviewed in [21,47]). However, males have been reported to resume swarming after mating. Therefore it may be possible for males to achieve a second mating on the same evening. This would mean that males might not be investing in one large ejaculate that depletes their capacity, but several smaller ones to ensure that they may mate with quality females. Indeed, laboratory reared *An. gambiae *are capable of inseminating up to five females, of which two included sperm and mating plugs [48].

Mating without swarming has been reported in several anophelines. Mating in alternate venues is often employed by individuals who are competitively disadvantaged [22]. As in other insect groups, smaller males or males who are physiologically stressed and not capable of swarming for long periods of time may adopt alternate mating strategies to ensure they pass on their genes [22]. Since the male antennal fibrillae become erect one hour before swarming commences, males should be sexually responsive to females in the vicinity even when not swarming ([23] and reported in [34]). Alternate venues may also favour sexual choice by allowing individuals a chance to select mates based on phenotypic qualities. Since females are considered to be monogamous, pre-copulatory choice would prevent mating with lesser quality males.

Once mating is completed, the female is assumed to become refractory to further mating due to the transfer of several male compounds. After ejaculation, males insert a mating plug composed of MAG substances [49]. In other culicids, MAG substances are responsible for causing the switch of female behaviour from unmated to mated. This was originally reinforced by reported polyandry in *An. gambiae s.l. *due to the inability of hybrid males with abnormal MAGs to prevent remating [50]. However, in the laboratory it was determined that the presence of sperm in the spermatheca is responsible for the switchover state in anophelines [51,52]. Though the function of MAG proteins in anophelines is unknown, several male-specific accessory gland proteins that are known to trigger post mating behavioural changes in *Drosophila *[49] have recently been identified in *An. gambiae*. Similarly, the insertion of the mating plug, and its associated substances, has been shown to turn off the mating machinery in *An. gambiae *females possibly resulting in a physiological barrier to remating [53]. Although superficially contradictory to earlier work [49,50], the processing of the MAG substances within the female's atrium may be necessary to stimulate post-mating processes, which might have been inadvertently by-passed in the original experiments [51].

The number of matings occurring in the swarm declines until darkness when most males disperse to rest and replenish energy reserves through nectar feeding. Males were found to expend up to 50% of their energy reserves during swarming and to feed shortly before dawn [54]. This meal was used mainly to fuel flight during the period prior to scotophase [55]. Nocturnal feeding, therefore, is required by males to replenish energy reserves utilized during mate seeking as well as for daily survival.

It has been argued that swarming is ritualistic and not important in mating (reviewed in [20]). Swarming without mating [23,28] or infrequent pairing [23,28,32,34] has been observed for many species. However, a great number of copulating pairs have been observed in swarms of these same species [31,39]. The crepuscular nature of swarming often hinders the researcher's ability to observe actual mating. Similarly, the number of sexually mature males far outnumbers the number of virgin females on any given evening [34]. This would result in an underestimation of the actual number of matings. Swarming may still be the most efficient way for males and females to meet in highly dispersed populations.

It is, therefore, unknown what male or female phenotypic characters, if any, are associated with mating success. Similarly, of the males that do mate, it remains unknown whether they copulate with several females to increase their chance of securing a high quality female (i.e. cryptic male choice).

## Hindrances to male mating

After sexual maturation, opportunities for a male to mate are limited by several factors. First, sugar feeding is important to both male survival and mating ability. Due to their small energy reserves relative to females, teneral males must locate a carbohydrate source in order to survive to sexual maturity [56]. Mortality in unfed *An. gambiae *males was reported to start at 24-29 h post emergence with 82% deceased within 48 h [57]. Concordant with field observations, males fed throughout the night in the laboratory and rested without imbibing during the day [58]. Not every male will feed every night; therefore the ability of a male to successfully locate a nectar source will be linked to its ability to swarm and mate [55].

Mating opportunities for older males may not occur either due to predation or diminished mating abilities. Colonised anophelines lived up to 20 days but based on recapture experiments it was estimated that the average lifespan of wild males was between 5-10 days [59,60] of which the first 1-2 days are devoted to sexual maturation. Although older males could represent a more genetically fit population [61], females mated to six day old males were less likely to oviposit than those mated to two day old males in the laboratory [15]. However, Reisen [62] found that there were similar numbers of mated and unmated males in both the 0-5 days and 5 day plus age groups. Reluctance for older males to mate, even though they still have spermatozoa in their testes, may be behavioural in nature and not a result of senescence [15,24]. In conclusion, it is unknown if older males are more or less likely to mate than their younger counterparts.

Next, not every male will have an opportunity to mate during its lifetime. In *An. stephensi *only 42% of males copulated in the laboratory [13]. Based on male sex organ dissections, ejaculation rates in wild male *An. culicifacies *varied from 14-47% with unmated individuals found in all age groups [62]. Smaller male *An. freeborni *had less success than their larger counterparts in inseminating females in swarms [26]. Certain males may be more fit than others - be it size or age - allowing them to secure more matings during their lifetime while less fit individuals acquire none. Also, the ability of certain males to replenish spermatozoa and MAGs after mating may limit the number of copulations achieved [13,62]. Early swarming males are also prone to higher predation rates [32,63]. Finally, the number of sexually mature, virgin females will be less than the number of sexually mature males available nightly [23,35]. Therefore, disproportionate mating opportunities and abilities combined with a lack of sexually receptive females would result in failure for some males to successfully mate.

The role of male size has been studied with interesting but inconsistent results with regard to mating success in anophelines. In *An. freeborni*, larger males mated more successfully than their smaller counterparts [26]. Conversely, in *An. funestus *males that were collected during swarming and *in copula *were no larger than those collected from indoor resting sites [32]. In *An. gambiae *there is conflicting evidence regarding male and female size and its influence on mating success [64-67]. One would not expect male size to be an important factor in swarm-based mating. In a no-choice situation males cannot risk refusing a female in hopes of finding a better quality mate [46].

Anopheline male mating success, therefore, relies on several parameters whose values are scarcely known and only for a few species. Male size does have an effect on reproductive abilities in temperate anophelines. Its effects, however, are unknown in tropical species, which represents an area that is in need of further research. What is known from laboratory studies and wild observations is that sexual maturation requires 1-2 days and males older than seven days are typically reluctant to mate. Therefore, a normal anopheline male may accomplish between 0-3 matings in his lifetime.

## The effects of colonisation on the mating behaviour of males

Released males must be able to 1) disperse over considerable ranges, 2) locate appropriate mating venues, and 3) compete with feral males [4]. Quality control protocols in the insectary are used to ensure that males achieve a reasonable level of quality, at least in relation to efficient mass rearing [68,69]. However these same protocols often act antagonistically in regards to dispersal and mating of the males in the field. Colonisation of vectors often leads to the inadvertent or purposeful fixation of alleles, which affect mating behaviours. Even when efforts are made to retain either large numbers of vectors, introduce wild material to maintain polymorphism, or by rearing under semi-natural conditions, colonisation will lead to behavioural changes in the vector [70-72]. The unnatural environment of the insectary led to a quick fixation of alleles or behaviours that resulted in assortative mating between colonised and native populations which were evident within four generations [73].

The establishment of a laboratory colony typically results in a bottle-neck due to the selective pressures placed on individuals to adapt to the unnatural environment. Unless an out-breeding scheme is implemented, this is even more pronounced during the establishment of a genetic sexing strain or a specific transgenic strain in which only one founder individual will have the correct phenotype. Kaneshiro [74] hypothesized that these bottle-necked subpopulations have altered their mate selection standards in an effort to avoid extinction. Normal mating behaviours may not be possible within a cage due to space limitations. This results in shortened or modified mating behaviours, which may be less desirable in the wild. Colonised insects therefore may become less "choosy" in mate selection: colonised insects would copulate with both laboratory-derived and wild insects while wild insects would reject matings with laboratory insects due to their unnatural mating behaviours.

Standardized rearing protocols are necessary to produce insects of known quality, and the environmental parameters employed typically do not recreate field conditions. This may lead to confusion of the male in regards to when or where to mate. In *Cochliomyia hominivorax*, it was reported that laboratory released flies were active later in the day than wild flies which were more active in the morning resulting in decreased encounters between the two populations [75]. Conversely, released *Bactrocera cucurbitae *were found to be more active earlier in the day while wild flies were active later [76]. In *An. gambiae *it was found that light bursts or alterations to the LD period caused disruptions in male flight activity [29,77]. *An. culicifacies *reared under standardized conditions failed to swarm properly in the wild for two days post-release [78], but swarming and mating were synchronized with wild mosquitoes when this strain was reared under a more natural photoperiod. In the laboratory, mating success of wild mosquitoes required both proper light intensity and the presence of a swarm marker while laboratory colonies mated indiscriminately [79].

Additionally, geographically distinct populations may exhibit different mating abilities. The accumulation of genetic differences necessary for survival and reproduction in different niches leads to ecological divergence and can result in pre-zygotic isolation [80]. Although geographic mating polymorphism has been reported between three colonies of *An. stephensi *[13,81], it has not been found to be a limiting factor in mating in *An. arabiensis*[82]. A population of *An. arabiensis *from La Réunion Island mated freely with continental African colonies. Therefore, efforts to discern the effects of geographic barriers to mating between disparate populations, as done in the Mediterranean fruit fly *Ceratitis capitata *SIT programme, would be necessary to ensure any colony established does not suffer from assortative mating [83].

Finally, colonisation has been associated with assortative mating behaviours. Colonised *Cx. tarsalis *released into an isolated area swarmed in different arenas compared to wild vectors [84]. Reisen [8] noted that even when a test population had a similar genetic background, assortative mating was still seen when released into the wild. It was concluded that assortative mating was the consequence of colonisation and that it occurred within 3-4 generations [73]. Even when released with laboratory reared, wild caught progeny, colonised vectors were discriminated against [85]. Chemosterilised *An. culicifacies *males were non-competitive in nature even though they rested and swarmed concomitantly with wild males and were equally competitive in laboratory trials [62]. Previous reports of assortative mating in released anophelines have involved long-colonised strains [62,86,87]. However, as reported by Kaiser *et al. *[88] and Helinski *et al. *[89], wild females did not discriminate against released males from recently colonised lines.

## Conclusion

In the last decade, the role of male mating biology has been repeatedly mentioned as a major limitation to any mass release programmes of sterile insects [7,8,90,91]. The success in controlling other dipterans utilizing SIT has largely been due to the extensive study of male mating biology [92-94]. Therefore, a fundamental knowledge of how males aggregate, encounter females, and copulate as well as techniques to detect alterations in mating behaviours is necessary.

Extensive field-testing is necessary to determine the level of mating competence between colonised vectors and native populations. Entraining colonised vectors to the local environment may increase success. Exposing teneral males to chemical or environmental cues may help condition them to locate sites where females are known to feed or mate. Males may locate mating arenas through experiences gained during their sexually immature period: site fidelity and memory have been suggested in dipterans such as *An. arabiensis *[95]. Teneral colony males could be housed near potential release sites. This would expose males to local parameters such as photoperiod, nectar sources, and environmental conditions to ensure they are capable of survival after release. The filter rearing system employed in the *C. capitata *SIT programme exposes colonised insects to more natural environmental conditions, natural host plants, and more natural rearing parameters resulting in a higher quality mating strain [68,96].

Lastly, is it possible to maintain a genetically modified colony long-term without detriment to mating behaviours? Colonisation of insect vectors for control programmes often leads to a "paradox of genetic breeding": the higher the output typically the lower the quality [97]. Although success in both medfly and melon fly SIT programmes have been attributed to long term colonisation without diminished mating abilities [93,96], planning should be undertaken to optimize rearing conditions with minimal alteration to "normal" abilities or behaviours. Optimization of rearing conditions, however, should be done with great caution so inadvertent selection of non-beneficial phenotypes is avoided.

### Key research questions

Before implementing an SIT programme, there are several points that need further research in order to increase the likelihood of success, especially in regard to *An. arabiensis*.

• Which phenotypic characters are associated with male mating success?

• Do males allocate all of their resources into one mating or instead invest in several smaller ejaculates?

• Do males swarm every night of their lives?

• Which mating behaviours are altered during colonisation and can these be avoided or their effects lessened?

• Will entraining vectors to local conditions prior to release increase their mating success?

• Will the geographic origin of the mosquito lead to assortative mating?

• How can we optimize rearing conditions to make a more competitive mosquito?

## Competing interests

The authors declare that they have no competing interests.

## Authors' contributions

PIH wrote the manuscript. BGJK critiqued and revised all sections. Both authors read and approved the final manuscript.
